# Editorial: Translational immunology in trauma - To provide new insights for improving outcomes

**DOI:** 10.3389/fmed.2022.1118290

**Published:** 2022-12-19

**Authors:** Klemens Horst, Ingo Marzi, Luke Leenen, Frank Hildebrand

**Affiliations:** ^1^Department of Orthopaedics, Traumatology and Reconstructive Surgery, Rheinisch Westfälische Technische Hochschule (RWTH) Aachen University, Aachen, Germany; ^2^Department of Trauma, Hand and Reconstructive Surgery, Goethe University Frankfurt, Frankfurt, Germany; ^3^Department of Trauma Surgery, University Medical Centre Utrecht, Utrecht, Netherlands

**Keywords:** translational, polytrauma, multiple trauma, research, severe injury, approach, outcome

As the importance of the post-traumatic and/or post-surgical immune response for outcomes is beyond any doubt, numerous pre-clinical experiments have focused on the underlying mechanisms. Thereby, both detrimental but also regenerative roles of immunological mediators have been described under these conditions. Both the complexity and unrevealed interactions of the immunologic response after trauma and surgery require interprofessional scientific exchange to share and connect new findings. Therefore, the articles included in this Research Topic reflect the current knowledge from latest basic science and clinical studies as well as reviews written by experts in their field.

Focusing on the central nervous system (CNS), Dobson et al. in their review outlined the molecular drivers of secondary injury, which include damage-associated molecular patterns (DAMPs), pathogen-associated molecular patterns (PAMPs) and other immune-modifying agents that activate the hypothalamic-pituitary-adrenal (HPA) axis and sympathetic stress response. Despite the potential limitations of previous studies [i.e., heterogeneity of humans, poorly designed trials, inappropriate use of specific pathogen-free (SPF) animals, ignoring sex-specific differences, and the flawed practice of single-nodal targeting] in studying drugs that target specific inflammatory pathways, the authors conclude that especially CNS protection and control of cardiovascular function maintain mitochondrial energetics, thereby resolving inflammation and minimizing immune dysfunction (Dobson et al.). Also focusing on the CNS, Ning et al. performed a single-center, prospective, randomized, and double-blind study on scalp nerve blockade (SNB) in pediatric patients undergoing craniotomy to investigate the effect on postoperative pain, intraoperative hemodynamic stability, and narcotic consumption under general anesthesia. The authors found adequate postoperative pain control and good intraoperative hemodynamic stability during noxious events in the intervention group compared to the control group (Ning et al.).

Focusing on immune dysfunction after trauma, Mizugaki et al. investigated the role of different neutrophil phenotypes in a murine burn-associated sepsis model. The group reported on higher levels of transforming growth factor-beta 1 and lower concentrations of cytokines in the burned compared to the sham group. Moreover, diverse neutrophil phenotypes (DC172a, CD68, CD11b) were observed to be differently expressed between the groups, suggesting that neutrophils are significantly involved in the immune imbalance following trauma (Mizugaki et al.). Wan et al. investigated the role and molecular mechanisms of Bruton's tyrosine kinase (BTK), a member of the Tec family in burn sepsis-induced intestinal injury. The authors found that LFM-A13 administration significantly inhibited p-BTK expression in the intestine. LFM-A13 also reduced the histopathological changes and cellular apoptosis in intestinal tissues, inhibited the release of pro- and anti-inflammatory cytokines (IL-4, IL-6, and TNF-alpha) in serum and intestinal tissues, and significantly inhibited the increase in intestinal MPO activity induced by burn-associated sepsis. The authors concluded that BTK activation is one important aspect of the signaling event that may mediate the release of pro- and anti-inflammatory cytokines, oxidative stress, and intestinal cell apoptosis and thus might contribute to burn sepsis-induced intestinal injury (Wan et al.).

The posttraumatic immune response after multiple trauma without an associated burn injury was investigated by Vollrath et al. The authors focused on the pathogen-specific reaction of phagocytes taken from a porcine polytrauma model at different time points, stimulated by different pathogens (i.e., *E. coli, S. aureus*, or *S. cerevisiae*). Throughout the observation phase of 72 h, the phagocytic activity and capacity of granulocytes and monocytes followed a significantly different pattern, depending on the pathogen strain (Vollrath et al.).

Xu et al. reports on Indoleamine 2,3-dioxygenase (IDO), its immune suppressive impact and potential in the emerging field of isolated liver injuries. As IDO is one of the initial rate-limiting enzymes of the kynurenine pathway (KP), causing immune suppression and the induction of T cell anergy, it is well-known to be associated with the imbalance of immune homeostasis and liver dysfunction (Xu et al.). In a comprehensive review, the authors give an overview of its function, impact, current research directions, and possible ways to use IDO in patients with hepatic damage. As liver dysfunction furthermore contributes negatively to the patients' coagulation system, Egea-Guerrero and Quintana-Diaz outline the role of prothrombin complex concentrate (PCC) in the severely injured patient and indicates careful application of PCC in the traumatized patient. Therefore, the authors point out that fibrinolysis must be controlled simultaneously to balance management of patients with hemorrhagic shock or trauma induced coagulopathy (Egea-Guerrero and Quintana-Diaz).

Cell function and characteristics are of highest importance to understand their involvement in the development of infectious complications (de Fraiture et al.). Analysis of neutrophil phenotypes and their function as complex biomarkers has become accessible for point-of-care decision making after trauma. Thus, potential neutrophil based cellular diagnostics may help to recognize patients at risk for infectious complications when presented in the trauma bay. These patients display increased numbers of neutrophil subsets, decreased responsiveness to fMLF and/or increased CD64 expression. According to these possibilities, de Fraiture et al. emphasizes measuring these biomarkers over time in patients at risk of infectious complications. This might help to guide an individual treatment strategy regarding the timing, extent of surgery and administration of (preventive) antibiotics (de Fraiture et al.). As complications significantly influence patient outcomes over the clinical course, Schindler et al. examined microbiological aspects of anti-infective treatment in trauma patients. Of 114 trauma patients, 45 suffered from post-traumatic infections during the first 10 days of hospitalization. Severely injured patients with concomitant traumatic brain injury (PT + TBI) showed the highest rate of post-traumatic infection. The leading entity of infection was pneumonia, followed by infections of the urinary tract. Although 67.5% of all trauma patients received single-shot antibiosis during initial care in the trauma bay, the development of secondary colonization was not relevantly positively correlated with single-shot antibiosis and prophylactically calculated antibiotic administration. Due to these findings, the authors concluded that one-time antibiotic and calculated prophylactic use of antibiotics, like cephalosporins, must be critically discussed in terms of their role in the development of post-traumatic infections and microbial selection (Schindler et al.).

In conclusion, this Research Topic presents the newest scientific insights on immunological and cellular reactions as well as tissue-related and circulatory responses after severe trauma or during the further clinical course of critically ill patients. This highly relevant data will deepen the understanding of the pathomechanistic influence of several *in vivo* and *in vitro* elaborated mediators and thus has the potential to improve the treatment of patients after a traumatic or surgical impact. We thank all the authors and the Publication Committee of *Frontiers in Medicine* for the privilege of publishing this Research Topic.

## Author contributions

KH, IM, LL, and FH defined the topic of this Research Topic. All authors contributed to the article and approved the submitted version.

